# Assessment of drug-related migraine in a real-world large-scale database

**DOI:** 10.3389/fphar.2025.1647088

**Published:** 2025-07-25

**Authors:** Fan Wu, Ao Liu, Zhenyuan Jiang, Zhonglin Wang

**Affiliations:** ^1^ Shandong University of Traditional Chinese Medicine, Jinan, Shandong, China; ^2^ Shanghai Academy of Social Sciences, Shanghai, China; ^3^ Affiliated Hospital of Shandong University of Traditional Chinese Medicine, Jinan, Shandong, China

**Keywords:** migraine, drug-induced headache, FAERS, pharmacovigilance, disproportionality analysis, BCPNN

## Abstract

**Background:**

Drug-induced migraine represents a clinically significant yet under-investigated subtype of migraine. This study aims to evaluate the risk of drug-related migraine based on real-world data from the FDA Adverse Event Reporting System (FAERS).

**Methods:**

A retrospective pharmacovigilance analysis was conducted using FAERS data from Q1 2004 to Q4 2024. Migraine cases were identified via standardized MedDRA (The Medical Dictionary for Regulatory Activities) terms. Only primary suspect drugs were included. Disproportionality analyses were performed using four algorithms: ROR, PRR, MGPS, and BCPNN. Drugs were classified by therapeutic indication and mechanism of action, and stratified by BCPNN values to assess risk levels.

**Results:**

A total of 20,886 migraine-related adverse events were identified, predominantly among females (77.4%) with a mean age of 45.7 years. Sixty-six drugs yielded positive signals, and after exclusion criteria, 39 remained for further analysis. The highest-risk agents included lorcaserin (BCPNN = 3.33), tasimelteon (3.20), and botulinum toxin type A (3.06). High-risk therapeutic classes included immunosuppressants, estrogens/progestogens, and sedative-hypnotics.

**Conclusion:**

This large-scale analysis identifies key drug categories and compounds associated with an elevated risk of migraine, providing actionable insights for clinicians. Especially lorcaserin, tasimelteon, and botulinum toxin as potential risk factors for migraine. Given the public health burden of migraine, pharmacovigilance efforts should incorporate such findings to mitigate iatrogenic risks. Further prospective studies are warranted to establish causal mechanisms and optimize therapeutic decision-making.

## Introduction

Migraine is characterized as a primary, episodic headache disorder distinguished by various combinations of autonomic nervous changes (Silberstein, 2004). The Global Burden of Disease Study categorizes migraine as the second most common neurological disorder worldwide (Chaturvedi et al., 2022), and the World Health Organization ranks it as the sixth leading cause of disability globally (Steiner et al., 2015). Moreover, increasing evidence suggests that migraine is a significant risk factor for acute cerebrovascular diseases (Etminan et al., 2005).

The repeated activation and subsequent sensitization of the trigeminovascular pathway are believed to lead to the occurrence of migraine (Burstein et al., 2010; Moskowitz and Macfarlane, 1993; Burstein et al., 1996), a phenomenon known as the trigeminovascular theory, which currently represents the predominant hypothesis for the pathogenesis of migraine. A key mechanism in the sensitization of the trigeminovascular system is neurogenic sterile meningeal inflammation (Moskowitz and Macfarlane, 1993): substantial evidence indicates that this neurogenic inflammation is likely induced by the release of sensory neuropeptides such as substance P and CGRP from innervating fibers (Waeber and Moskowitz, 2005; Lundberg et al., 1984). These peptides typically cause vasodilation, plasma protein extravasation, and local activation of mast cells in the dura mater, leading to the release of cytokines and other inflammatory mediators, which in turn trigger neurogenic inflammation (Moskowitz and Macfarlane, 1993). Throughout the phases of a migraine attack, vascular and neural elements interact in a complex manner (Aurora and Chronicle, 2002). In addition to well-recognized factors such as familial genetics, emotional fluctuations, and external stimuli, the use of various medications also serves as a significant trigger for secondary migraine.

Pharmaceutical agents widely recognized as potent triggers for migraine include hormonal treatments, nitric oxide donors, and other substances known to provoke migraines (Silberstein and Merriam, 1993; Olesen et al., 1995), as well as oral contraceptives (Cupini et al., 1995). Despite the prevalence of these triggers, there is a significant lack of intuitive, data-driven analytical studies in this field. Presently, even multicentric clinical trials suffer from inadequate resources in terms of both personnel and funding to maintain extensive, longitudinal records. The reliance on a limited number of researchers to elucidate the complex clinical effects of various drugs does not provide a sufficient efficacy-to-cost ratio. In this context, the FDA Adverse Event Reporting System (FAERS) database, as the largest global repository of adverse event reports, amalgamates data from manufacturers with voluntary reports submitted by healthcare providers and the public via the MedWatch program (Sakaeda et al., 2013). This integration offers substantial benefits. Currently, the exploitation of real-world data from the FAERS database to investigate and delineate the risk characteristics of pharmaceuticals has become a critical approach for evaluating drug safety (Beninger, 2018; Lucas et al., 2022). As a comprehensive record of clinical events devoid of conflicts of interest, these data uniquely benefit from authenticity and lack of bias inherent in their randomness and reality. To some extent, this transcends the inherent limitations related to scale, duration, and selection of population that are typical of preclinical studies and provides a cost-effective, real-time overview of primary toxicities, thereby informing clinical practice (Li et al., 2023). Consequently, this study aims to comprehensively assess the risk factors associated with drug-induced migraines and to conduct a thorough investigation of these migraines using the FAERS database, exploring potential risk factors.

## Methods

### Data sources

**FIGURE 1:**
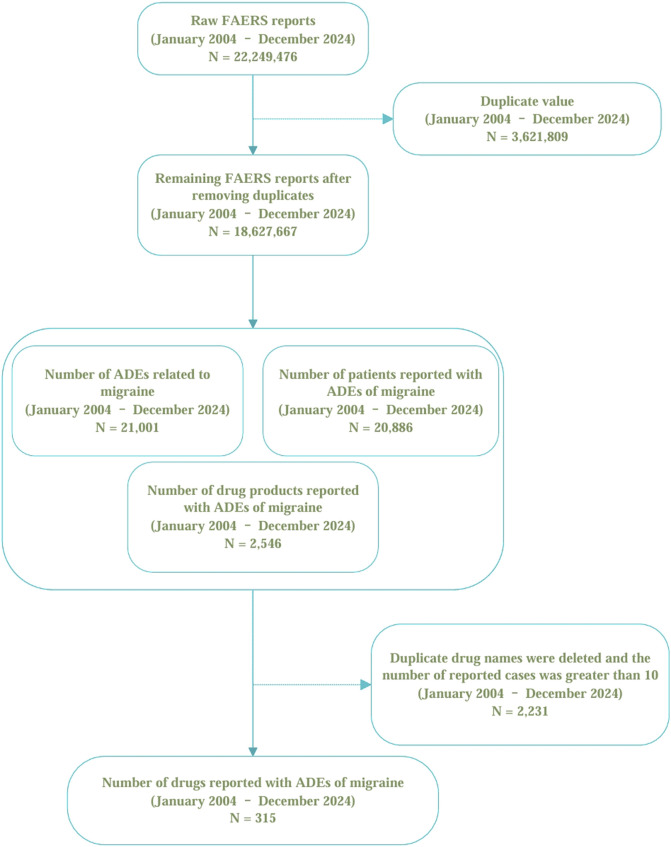
The selection of migraine-related cases and the data cleaning process in the FAERS database.

For this retrospective pharmacovigilance analysis, we utilized the FAERS database, which supports the FDA’s post-marketing safety surveillance program for all marketed drug and therapeutic biologic products. The database comprises seven datasets: patient demographics and administrative information (DEMO), drug and biologic information (DRUG), adverse events (REAC), patient outcomes (OUTC), report sources (RPSR), start and end dates of drug therapy (THER), and drug use and diagnostic indications (INDI) (Sakaeda et al., 2013). The FAERS database, accessible via the FDA’s official website (https://www.fda.gov/drugs/drug-approvals-and-databases/fda-adverse-event-reporting-system-faers-database), publicly discloses all AE reports received since 2004. The data is anonymized by omitting personal identifiers and assigning a unique code (‘primaryid'), thus ensuring that individuals recorded in the database cannot be identified. Consequently, this study conforms to ethical standards and does not necessitate IRB approval, aligning with FDA policies on data privacy and confidentiality (Moreland-Head et al., 2021). Our study data were selected from the first quarter of 2004 through the fourth quarter of 2024, encompassing all migraine-related adverse events recorded by the FDA. From a total of 22,249,476 original data entries collected between these dates, after the deletion of duplicates, 18,627,667 entries remained. A total of 21,001 literature reports documented adverse events related to migraine, affecting 20,886 subjects. There were 2,546 drugs associated with migraine adverse reactions. Due to duplication of commercial brand names, drugs reported in fewer than ten cases were excluded, this cutoff minimizes spurious signals from small samples (Carnovale et al., 2018), leaving 315 unique drugs after duplicates were removed (see Figure 1).

### Identification of adverse reactions

In this analysis, the definition of adverse drug reactions (ADRs) adheres to the guidelines set forth in the Medical Dictionary for Regulatory Activities (MedDRA, http://www.meddra.org/) Version 20.0 (Brown et al., 1999). Adverse events were coded using MedDRA preferred terms (PTs), and standardized MedDRA queries were employed to identify PTs related to migraine. This study specifically utilized “narrow” scope PTs (Mozzicato, 2007).

### Data extraction

In this study, the incidence of migraine reports was identified in the RACE file utilizing the preferred term “migraine” along with their associated primaryid codes. These primaryids were then employed to eliminate duplicate reports in the DEMO file, thereby accurately determining the number of migraine cases. Within the FAERS database, drugs are categorized into four groups: “Primary Suspect Drug,” “Secondary Suspect Drug,” “Concomitant Drug,” and “Interacting Drug.” To mitigate the uncertainty associated with the correlation between certain drugs and adverse reactions, this research exclusively utilized data pertaining to drugs identified solely as the Primary Suspect (PS) causing the adverse reaction, excluding those classified as “Secondary Suspect Drugs,” “Concomitant Drugs,” and “Interacting Drugs.”

### Statistical analysis

To minimize bias and identify potential signals of adverse events, this research employed four distinct disproportionality analysis methods: the Reporting Odds Ratio (ROR) (Rothman et al., 2004), the Proportional Reporting Ratio (PRR) (Evans et al., 2001), the Bayesian Confidence Propagation Neural Network (BCPNN) (Bate, 2007), and the Multi-item Gamma Poisson Shrinker (MGPS) (Berlin et al., 2012). Each method was conducted using signal detection parameters derived from a 2x2 contingency table, as shown in Table 1. Detailed formulas and criteria for signal generation are presented in Table 2. The criteria for positive signal detection are detailed in Table 2 (Wu et al., 2025; Wu et al., 2024). In this analysis, a drug was only considered to have a potential association with an event if it yielded positive results across all four algorithms. Subsequently, the BCPNN algorithm was utilized to stratify the risk of migraine associated with different drugs.

**TABLE 1:** Four-grid table of disproportionality analysis method.

Item	Target adverse events	All other adverse events	Total
Target drugs	a	b	a+b
All other drugs	c	d	c + d
Total	a+c	b + d	a+b + c + d

Notes: A contingency table for the calculation formula of the proportion imbalance analysis.

**TABLE 2:** Principle of disproportionality analysis and standard of signal detection.

Methods	Calculation formula	Inclusion standard of positive signal
ROR	$\text{ROR}=\frac{\left(a/c\right)}{\left(b/d\right)}$	a≥3 and 95%CI > 1
	$\text{SE}\left(\ln\text{ROR}\right)=\sqrt{\left(\frac{1}{a}+\frac{1}{b}+\frac{1}{c}+\frac{1}{d}\right)}$	
	$95\%\text{CI}=e^{\ln\left(\text{ROR}\right)\pm 1.96\sqrt{\frac{1}{a}+\frac{1}{b}+\frac{1}{c}+\frac{1}{d}}}$	
PRR	$\text{PRR}=\frac{a/\left(a+b\right)}{c/\left(c+d\right)}$	a≥3 and 95%CI > 1
	$\text{SE}\left(\ln\text{PRR}\right)=\sqrt{\left(\frac{1}{a}-\frac{1}{a+b}+\frac{1}{c}-\frac{1}{c+d}\right)}$	
	$95\%\text{CI}=e^{\ln\left(\text{PRR}\right)\pm 1.96\sqrt{\left(\frac{1}{a}-\frac{1}{a+b}+\frac{1}{c}-\frac{1}{c+d}\right)}}$	
BCPNN	$\text{IC}=\log_{2}\frac{a\left(a+b+c+d\right)}{\left(a+b\right)\left(a+c\right)}$	1) No Signal (−): IC_025_ ≤ 0 2) Low Signal (+):0<IC_025_ ≤ 1.5 3) Medium Signal (++):1.5<IC_025_ ≤ 3 4) High Signal (+++): IC_025_ > 3
	${\text{IC}}_{025}=\log_{2}\frac{\left(a+\gamma 11\right)\left(a+b+c+d+\alpha \right)\left(a+b+c+d+\beta \right)}{\left(a+b+c+d+\gamma \right)\left(a+b+\alpha 1\right)\left(a+c+\beta 1\right)}$	
	$V\left(\text{IC}\right)=\frac{1}{{\left(\ln2\right)}^{2}}\left\{\left[\frac{\left(a+b+c+d\right)-a+\gamma -\gamma 11}{\left(a+\gamma 11\right)\left(1+a+b+c+d+\gamma \right)}\right]+\left[\frac{\left(a+b+c+d\right)-\left(a+b\right)+a-\alpha 1}{\left(a+b+\alpha 1\right)\left(1+a+b+c+d+\alpha \right)}\right]+\left[\frac{\left(a+b+c+d\right)-\left(a+c\right)+\beta -\beta 1}{\left(a+c+\beta 1\right)\left(1+a+b+c+d+\beta \right)}\right]\right\}$	
	$\gamma =\gamma 11\frac{\left(a+b+c+d+\alpha \right)\left(a+b+c+d+\beta \right)}{\left(a+b+\alpha 1\right)\left(a+c+\beta 1\right)}$	
	$\text{IC}-2\text{SD}=E\left(\text{IC}\right)-2\sqrt{V\left(\text{IC}\right)}$	
	Where α1 = β1 = 1; α = β = 2; γ11=1	
MGPS	$\text{EBGM}=\frac{a\left(a+b+c+d\right)}{\left(a+c\right)\left(a+b\right)}$	EBGM05 > 2 and a>0
	$\text{EBGM}05=e^{\ln\left(\text{EBGM}\right)-\sqrt[{2}]{1.64\left(\frac{1}{a}+\frac{1}{b}+\frac{1}{c}+\frac{1}{d}\right)}}$	

Abbreviation: ROR, reporting odds ratio; PRR, proportional reported ratio; BCPNN, bayesian confidence propagation neural network; MGPS, multi-item gamma poisson shrinker; CI, confidence interval; IC, information component.

## Result

### Descriptive analysis of subjects

**FIGURE 2:**
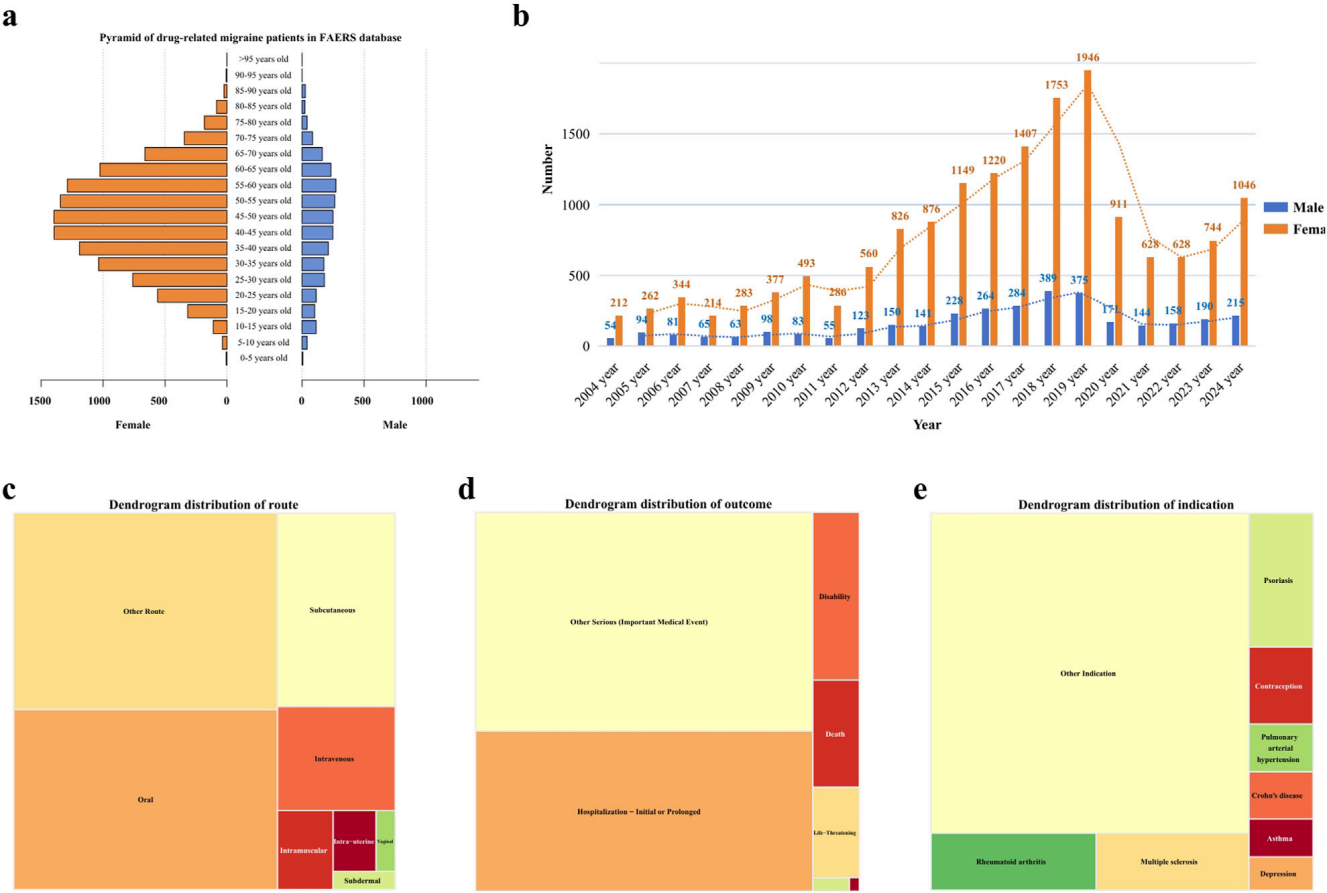
Distribution of baseline data for patients reporting migraine-related adverse events in the FAERS database. **(a)** Age distribution pyramid by gender for patients reporting migraine-related adverse events. **(b)** Temporal distribution of migraine-related adverse event reports. **(c)** Distribution of routes of administration for migraine-related adverse events. **(d)** Histogram of outcomes for patients with migraine-related adverse events. **(e)** Distribution of drug use causing migraine-related adverse events.

This study included a total of 20,886 participants who reported migraine-related adverse events. The mean age of the cohort was 45.74 ± 15.70 years, with a predominance of female patients (77.40%) over male patients (16.40%). This gender disparity was observed across all age groups. The majority of adverse events occurred in individuals aged between 20 and 70 years (see Figure 2a). From 2004 to 2019, there was a steady increase in the number of migraine cases reported in the FAERS database, reaching a peak in 2019, followed by a sharp decline through 2021, and a subsequent steady increase from 2021 to 2024. Overall, the trend from 2004 to 2024 remained upward (see Figure 2b). Regarding the distribution of reporters by profession, physicians constituted the largest group, comprising 12,118 individuals (58.02%), followed by other healthcare professionals, numbering 6,062 (29.02%) (see Figure 2c). The most common routes of administration for drugs associated with adverse events were oral (32.99%), followed by subcutaneous injection (15.88%), and intravenous injection (8.57%) (see Figure 2d). In terms of patient outcomes, the majority involved “other serious conditions” (28.84%), with hospitalization being the second most common outcome (21.12%) (see Figure 2e). Geographically, the United States reported the highest number of cases (14,746 cases; 70.60%), followed by Canada (9.49%), the United Kingdom (5.34%), and Germany (2.18%). Further demographic details are available in Table 3.

**TABLE 3:** Baseline characteristics of drug-associated migraine cases.

Variable	Formula	Total
Age	Mean ± SD	45.74 ± 15.70
Weight	Mean ± SD	76.56 ± 23.55
Gender		
Female	n (%)	16,165 (77.40)
Male	n (%)	3,425 (16.40)
Unknown	n (%)	1,296 (6.21)
Reporter		
Physician	n (%)	12,118 (58.02)
Other health-professional	n (%)	6,062 (29.02)
Pharmacist	n (%)	2,706 (12.96)
Country		
United states	n (%)	14,746 (70.60)
Canada	n (%)	1983 (9.49)
United kingdom	n (%)	1,116 (5.34)
Germany	n (%)	456 (2.18)
France	n (%)	444 (2.13)
Australia	n (%)	205 (0.98)
Italy	n (%)	133 (0.64)
Brazil	n (%)	117 (0.56)
Spain	n (%)	106 (0.51)
Netherlands	n (%)	85 (0.41)
Other country	n (%)	1,495 (7.16)
Route		
Oral	n (%)	6,891 (32.99)
Subcutaneous	n (%)	3,316 (15.88)
Intravenous	n (%)	1789 (8.57)
Intramuscular	n (%)	639 (3.06)
Intra-uterine	n (%)	381 (1.82)
Vaginal	n (%)	167 (0.80)
Subdermal	n (%)	163 (0.78)
Other route	n (%)	7,540 (36.10)
Outcome		
Other Serious (Important medical event)	n (%)	6,024 (28.84)
Hospitalization - Initial or Prolonged	n (%)	4,412 (21.12)
Disability	n (%)	636 (3.05)
Death	n (%)	407 (1.95)
Life-threatening	n (%)	344 (1.65)
Required Intervention to prevent permanen impairment/damage	n (%)	40 (0.19)
Congenital Anomaly	n (%)	11 (0.05)
Unknown	n (%)	9,012 (43.15)
Indication		
Rheumatoid arthritis	n (%)	1,364 (6.53)
Multiple sclerosis	n (%)	1,262 (6.04)
Psoriasis	n (%)	1,244 (5.96)
Contraception	n (%)	717 (3.43)
Pulmonary arterial hypertension	n (%)	443 (2.12)
Crohn’s disease	n (%)	438 (2.10)
Asthma	n (%)	355 (1.70)
Depression	n (%)	307 (1.47)
Other Indication	n (%)	14,756 (70.65)

Notes: Continuous variables are presented as mean ± standard deviation, and categorical variables are presented as n (%).

### Drug screening and disambiguation analysis

In the screening of 2,546 drugs, those with fewer than ten reported cases were excluded. A disambiguation analysis was then conducted on the remaining 315 drugs, each associated with ten or more case reports of drug-related migraine incidents, identifying 66 drugs with positive signals. Subsequently, the DrugBank database (DrugBank, 2024) was utilized to ascertain the brand names, generic names, and mechanisms of action for these 66 drugs. After excluding drugs with therapeutic effects on migraine and consolidating drugs under the same generic name, the list was narrowed down to 39 drugs. Their signal values were recalculated for further analysis.

### Classification of drugs by therapeutic purpose

**FIGURE 3:**
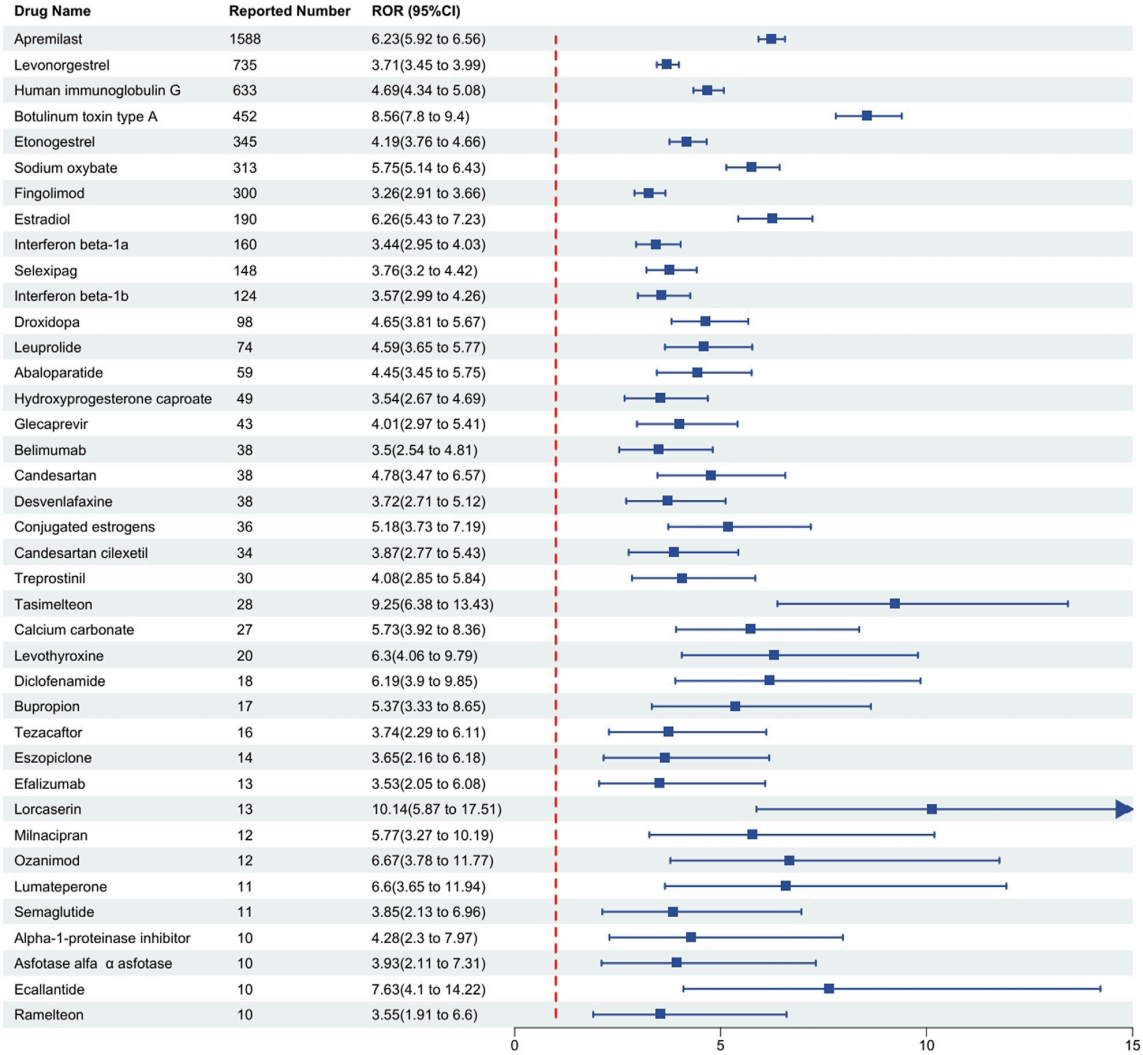
Detection of positive signal drugs through disproportionality analysis and their categorization based on mechanism of action. Notes: This figure classifies these positive signal drugs according to their mechanisms of action, where higher signal values indicate a greater risk of drug-related macular degeneration. The Reporting Odds Ratio (ROR) is presented as a measure of relative risk. Abbreviations: CI, confidence interval.

The drugs were categorized based on their therapeutic purposes. According to the number of reports, the top three categories were rheumatoid arthritis (6.53%), including Human immunoglobulin G (ROR = 4.69), Belimumab (ROR = 3.5); multiple sclerosis (6.04%), including Fingolimod (ROR = 3.26), Interferon beta-1a (ROR = 3.44), Interferon beta-1b (ROR = 3.57), Ozanimod (ROR = 6.67); and psoriasis (5.96%), including Apremilast (ROR = 6.23), Efalizumab (ROR = 3.53). Conversely, some therapeutic categories, though less prevalent in terms of case numbers, encompassed a rich variety of drugs. A typical example is contraception (3.43%), which included drugs such as Levonorgestrel (ROR = 3.71), Etonogestrel (ROR = 4.19), and Hydroxyprogesterone caproate (ROR = 3.54). Antidepressants (1.47%) comprised Desvenlafaxine (ROR = 3.72), Bupropion (ROR = 5.37), Milnacipran (ROR = 5.77), while medications for sleep disorders included Tasimelteon (ROR = 9.25), Eszopiclone (ROR = 3.65), and Ramelteon (ROR = 3.55). Medications for pulmonary arterial hypertension (2.12%) included Selexipag (ROR = 3.76) and Treprostinil (ROR = 4.08). Further details are illustrated in Figure 3 and Table 4.

**TABLE 4:** Disproportionality analysis: positive signals of drugs associated with migraine.

Drug name	ROR (95%CI)	PRR (95%CI)	MGPS (95%CI)	BCPNN (95%CI)	PRR (X^2^)	P Value
Apremilast	6.23 (5.92–6.56)	6.2 (6.15–6.25)	5.81 (5.56–6.06)	2.54 (0.87–4.2)	6.2 (6,411.37)	<0.001
Levonorgestrel	3.71 (3.45–3.99)	3.7 (3.63–3.77)	3.61 (3.39–3.84)	1.85 (0.18–3.52)	3.7 (1,399.16)	<0.001
Human immunoglobulin G	4.69 (4.34–5.08)	4.68 (4.6–4.76)	4.57 (4.27–4.88)	2.19 (0.52–3.86)	4.68 (1776.07)	<0.001
Botulinum toxin type A	8.56 (7.8–9.4)	8.5 (8.4–8.59)	8.34 (7.71–9.01)	3.06 (1.39–4.73)	8.5 (2,928.48)	<0.001
Etonogestrel	4.19 (3.76–4.66)	4.17 (4.07–4.28)	4.12 (3.77–4.51)	2.04 (0.38–3.71)	4.17 (820.09)	<0.001
Sodium oxybate	5.75 (5.14–6.43)	5.72 (5.61–5.83)	5.65 (5.14–6.2)	2.5 (0.83–4.16)	5.72 (1,201.97)	<0.001
Fingolimod	3.26 (2.91–3.66)	3.25 (3.14–3.37)	3.22 (2.93–3.54)	1.69 (0.02–3.35)	3.25 (462.07)	<0.001
Estradiol	6.26 (5.43–7.23)	6.23 (6.09–6.37)	6.18 (5.48–6.97)	2.63 (0.96–4.29)	6.23 (827.5)	<0.001
Interferon beta-1a	3.44 (2.95–4.03)	3.44 (3.28–3.59)	3.42 (3–3.89)	1.77 (0.11–3.44)	3.44 (274.54)	<0.001
Selexipag	3.76 (3.2–4.42)	3.75 (3.59–3.91)	3.73 (3.26–4.27)	1.9 (0.23–3.57)	3.75 (296.98)	<0.001
Interferon beta-1b	3.57 (2.99–4.26)	3.56 (3.39–3.74)	3.55 (3.06–4.11)	1.83 (0.16–3.49)	3.56 (227.49)	<0.001
Droxidopa	4.65 (3.81–5.67)	4.63 (4.43–4.83)	4.61 (3.9–5.45)	2.21 (0.54–3.87)	4.63 (277.8)	<0.001
Leuprolide	4.59 (3.65–5.77)	4.58 (4.35–4.8)	4.56 (3.77–5.53)	2.19 (0.52–3.86)	4.58 (206.33)	<0.001
Abaloparatide	4.45 (3.45–5.75)	4.44 (4.18–4.69)	4.43 (3.57–5.49)	2.15 (0.48–3.81)	4.44 (156.91)	<0.001
Hydroxyprogesterone caproate	3.54 (2.67–4.69)	3.53 (3.25–3.81)	3.53 (2.79–4.46)	1.82 (0.15–3.48)	3.53 (88.87)	<0.001
Glecaprevir	4.01 (2.97–5.41)	4 (3.7–4.3)	3.99 (3.11–5.13)	2 (0.33–3.66)	4 (96.62)	<0.001
Belimumab	3.5 (2.54–4.81)	3.49 (3.17–3.81)	3.49 (2.67–4.55)	1.8 (0.14–3.47)	3.49 (67.51)	<0.001
Candesartan	4.78 (3.47–6.57)	4.76 (4.44–5.08)	4.75 (3.64–6.21)	2.25 (0.58–3.92)	4.76 (112.77)	<0.001
Desvenlafaxine	3.72 (2.71–5.12)	3.71 (3.39–4.03)	3.71 (2.84–4.84)	1.89 (0.22–3.56)	3.71 (75.19)	<0.001
Conjugated estrogens	5.18 (3.73–7.19)	5.16 (4.83–5.48)	5.15 (3.91–6.78)	2.36 (0.7–4.03)	5.16 (120.56)	<0.001
Candesartan cilexetil	3.87 (2.77–5.43)	3.86 (3.53–4.2)	3.86 (2.91–5.12)	1.95 (0.28–3.61)	3.86 (72.11)	<0.001
Treprostinil	4.08 (2.85–5.84)	4.07 (3.71–4.42)	4.06 (3.01–5.48)	2.02 (0.36–3.69)	4.07 (69.34)	<0.001
Tasimelteon	9.25 (6.38–13.43)	9.18 (8.81–9.55)	9.17 (6.71–12.52)	3.2 (1.53–4.86)	9.18 (203.98)	<0.001
Calcium carbonate	5.73 (3.92–8.36)	5.7 (5.32–6.08)	5.69 (4.15–7.81)	2.51 (0.84–4.18)	5.7 (104.58)	<0.001
Levothyroxine	6.3 (4.06–9.79)	6.27 (5.83–6.71)	6.26 (4.34–9.05)	2.65 (0.98–4.31)	6.27 (88.59)	<0.001
Diclofenamide	6.19 (3.9–9.85)	6.16 (5.7–6.62)	6.16 (4.18–9.08)	2.62 (0.96–4.29)	6.16 (77.85)	<0.001
Bupropion	5.37 (3.33–8.65)	5.34 (4.87–5.82)	5.34 (3.58–7.96)	2.42 (0.75–4.08)	5.34 (60.04)	<0.001
Tezacaftor	3.74 (2.29–6.11)	3.73 (3.24–4.22)	3.73 (2.47–5.62)	1.9 (0.23–3.56)	3.73 (31.98)	<0.001
Eszopiclone	3.65 (2.16–6.18)	3.64 (3.12–4.17)	3.64 (2.35–5.65)	1.87 (0.2–3.53)	3.64 (26.88)	<0.001
Efalizumab	3.53 (2.05–6.08)	3.52 (2.98–4.06)	3.52 (2.23–5.55)	1.81 (0.15–3.48)	3.52 (23.44)	<0.001
Lorcaserin	10.14 (5.87–17.51)	10.04 (9.5–10.58)	10.04 (6.35–15.86)	3.33 (1.66–5)	10.04 (105.9)	<0.001
Milnacipran	5.77 (3.27–10.19)	5.75 (5.18–6.31)	5.74 (3.57–9.24)	2.52 (0.85–4.19)	5.75 (47.08)	<0.001
Ozanimod	6.67 (3.78–11.77)	6.63 (6.07–7.2)	6.63 (4.12–10.67)	2.73 (1.06–4.4)	6.63 (57.45)	<0.001
Lumateperone	6.6 (3.65–11.94)	6.56 (5.97–7.15)	6.56 (3.99–10.77)	2.71 (1.05–4.38)	6.56 (51.87)	<0.001
Semaglutide	3.85 (2.13–6.96)	3.84 (3.25–4.43)	3.84 (2.34–6.3)	1.94 (0.27–3.61)	3.84 (23.12)	<0.001
Alpha-1-proteinase inhibitor	4.28 (2.3–7.97)	4.27 (3.65–4.88)	4.26 (2.54–7.17)	2.09 (0.43–3.76)	4.27 (25.01)	<0.001
Asfotase alfa α asfotase	3.93 (2.11–7.31)	3.92 (3.3–4.53)	3.91 (2.33–6.58)	1.97 (0.3–3.64)	3.92 (21.72)	<0.001
Ecallantide	7.63 (4.1–14.22)	7.58 (6.97–8.2)	7.58 (4.5–12.76)	2.92 (1.25–4.59)	7.58 (57.18)	<0.001
Ramelteon	3.55 (1.91–6.6)	3.54 (2.92–4.16)	3.54 (2.1–5.95)	1.82 (0.16–3.49)	3.54 (18.23)	<0.001

Note: The p-value represents the statistical test value from the chi-square test in the PRR, algorithm. All of the above drugs meet the positive signal screening criteria for disproportionality analysis.

Abbreviations: BCPNN, bayesian confidence propagation neural network; MGPS, multi-item gamma Poisson shrinker; PRR, proportional reported ratio; ROR, reporting odds ratio; CI, confidence interval.

### Classification of drugs by mechanism of action

Drugs are grouped according to their different mechanisms of action based on the ATC classification in DRUGBANK. The classification by the degree of risk associated with adverse reactions reveals a descending order of drug categories: unclassified (17.95%), immunosuppressants (12.82%), estrogens and progestogens (10.26%), and sedative-hypnotics (10.26%), among others, as detailed in Table 5.

**TABLE 5:** Grouping of medications by different mechanisms of action based on the ATC classification from DRUGBANK.

Miscellaneous unclassified medications	Leuprolide
	Botulinum toxin type A
	Hydroxyprogesterone caproate
	Candesartan cilexetil
	Alpha-1-proteinase inhibitor
	α asfotase
	Ecallantide
Immunosuppressive agents	Apremilast
	Fingolimod
	Belimumab
	Efalizumab
	Ozanimod
Estrogens and progestogens	Levonorgestrel
	Etonogestrel
	Estradiol
	Conjugated estrogens
Immune enhancers	Human immunoglobulin G
	Interferon beta-1a
	Interferon beta-1b
Antidepressants	Desvenlafaxine
	Bupropion
	Milnacipran
Stabilizing agents	Eszopiclone
	Tasimelteon
	Lumateperone
	Ramelteon
Platelet aggregation inhibitors	Selexipag
	Treprostinil
Non-sexual hormonal medications	Abaloparatide
	Levothyroxine
Other neurological agents	Sodium oxybate
Adrenergic and dopaminergic medications	Droxidopa
Anti-obesity agents	Lorcaserin
Diabetes medications	Semaglutide
Antiviral medications	Glecaprevir
Angiotensin II receptor blockers	Candesartan
Calcium supplementation	Calcium carbonate
Carbonic anhydrase inhibitors	Diclofenamide
Respiratory system products	Tezacaftor

**FIGURE 4:**
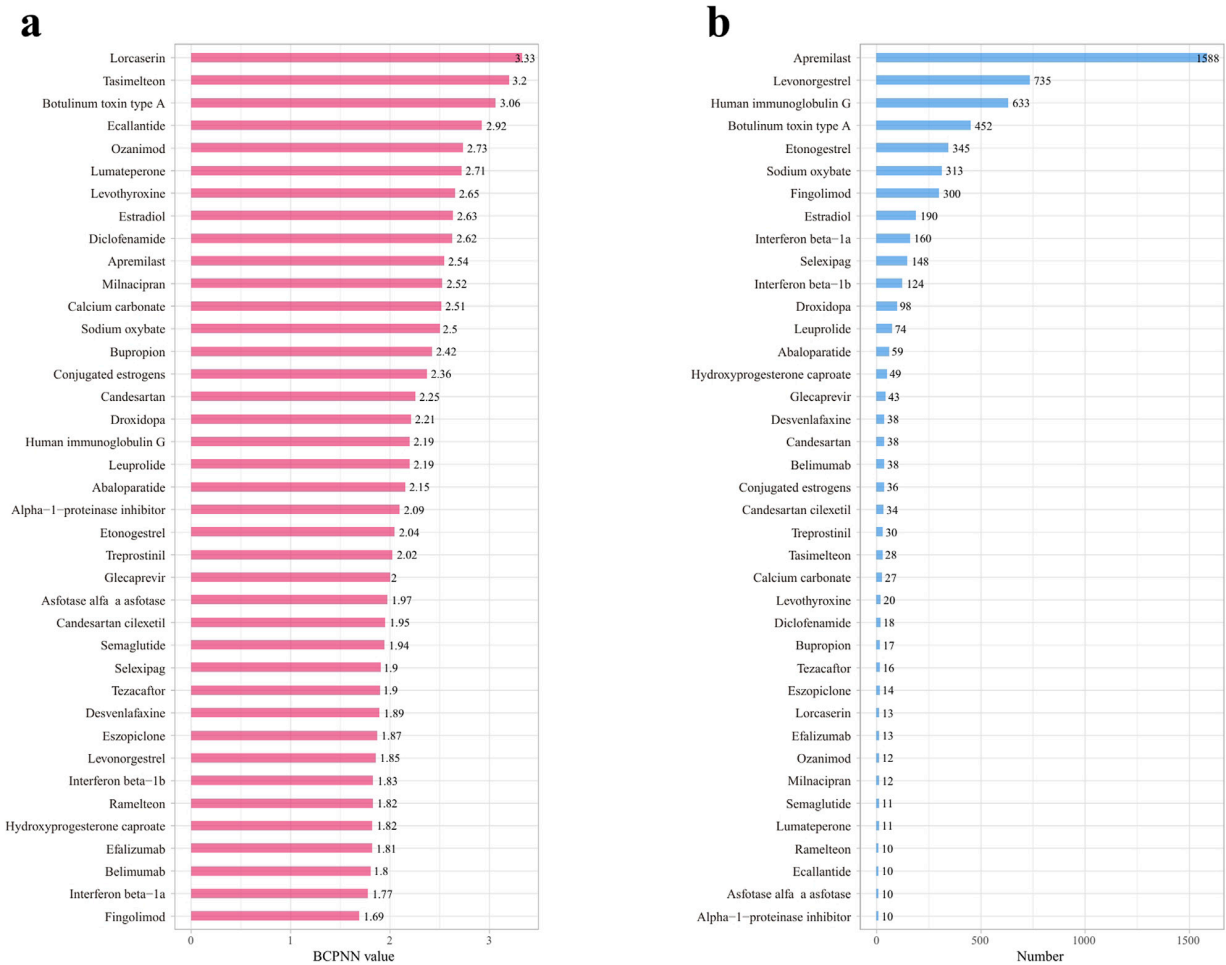
Risk levels and reporting volumes for drug-related migraine adverse reactions, sorted by decreasing risk and reporting volume. **(a)** Risk levels of positive drugs assessed using the BCPNN algorithm. **(b)** Reporting volumes for drug-related migraine adverse reactions, decreasing from high to low.

We employed the Bayesian Confidence Propagation Neural Network (BCPNN) algorithm to evaluate the risk of drug-related migraine. The BCPNN values categorize the risk levels as follows: values between 0 and 1.5 indicate low risk, between 1.5 and 3 signify moderate risk, and values above 3 denote high risk (Wu et al., 2025). Using these criteria, we assessed the migraine risk associated with 39 drugs. The three drugs with the highest risk were Lorcaserin (BCPNN = 3.33), Tasimelteon (BCPNN = 3.2), and Botulinum toxin type A (BCPNN = 3.06), with the remaining drugs classified as moderate risk, and no drugs falling into the low-risk category. Details are provided in Figure 4.

## Discussion

Migraine not only significantly impairs the quality of life for sufferers but also considerably increases their risk of experiencing a cerebral infarction. In this study, we systematically analyzed adverse events related to drug-induced migraine reported in the FAERS database since its inception in January 2004. Our findings indicate that drug-related migraine cases are predominantly reported among elderly women in Western countries. Employing four disproportionality analysis methods, we identified 39 drugs with positive signals for drug-related migraine. These medications span a variety of mechanisms of action and exhibit significant differences in their associated risks of inducing migraine. Notably, categories such as immunosuppressants, estrogens and progestogens, and antidepressants warrant particular attention due to their high association with adverse reactions to migraine. Drugs such as Lorcaserin, Tasimelteon, and Botulinum toxin type A, which are associated with the highest risks of adverse reactions, also warrant caution. These findings provide valuable real-world data and theoretical support for clinical decision-making, guiding the judicious use of medications to reduce the risk of migraine. They emphasize the necessity of careful consideration in clinical settings and offer significant insights for future research.

In the existing body of research, the incidence of migraine among female patients is typically three times that of their male counterparts (Stewart et al., 1992). This disparity appears to be linked to fluctuations in estrogen levels (Lipton et al., 2001; Somerville, 1975). Prior to puberty, the prevalence of migraine is approximately 4% and does not significantly differ between genders. However, post-puberty, the incidence among females not only increases more rapidly than in males but also peaks around the age of 40 before subsequently declining (Bigal et al., 2004). This study specifically examines medication-related migraine, a subtype of migraine, where the pattern described above is even more pronounced. Before the age of 15, the reported cases of migraine are nearly equal between genders, with males occasionally reporting slightly more cases. Nevertheless, following the onset of puberty, the number of migraine reports in females surges, continuing until around the age of 50, after which it begins to decrease. In this data, the ratio of female to male migraine reports significantly exceeds the 3:1 ratio, with the highest reaching between 4:1 and 5:1. Moreover, in this study, the incidence of new cases among males roughly follows a pattern of increasing post-puberty and then declining in middle age. While the pattern in females can be attributed to the regulatory effects of estrogen, current research does not adequately explain why the timing of onset in males follows a similar pattern.

In the findings of this study, reports of medication-related migraine, categorized as an adverse event, exhibited a consistent increase from 2004 to 2019. However, a marked decline occurred during 2020–2021, followed by a return to a gradual increase from 2021 onwards. Several factors may account for these trends. Firstly, during the COVID-19 pandemic, there was a frequent use of Renin-Angiotensin System (RAS) blockers and non-steroidal anti-inflammatory drugs (NSAIDs) (Li et al., 2003) such as ibuprofen, which are often used off-label for the prophylactic treatment of migraines (Loder and Rizzoli, 2018). This practice likely contributed to a reduction in migraine occurrences. Secondly, the increase in migraine cases due to other causes, which were often confused with medication-related migraines, could have led to a decrease in the reporting of medication-related adverse events. Specific factors include: (a) headache being one of the earliest and most common symptoms of COVID-19, affecting 14%–60% of patients in the early stages of the disease (Martelletti et al., 2020; Pullen et al., 2020); (b) COVID-19 primarily affects the respiratory system (Renda et al., 2022; Polverino et al., 2020; Tana et al., 2022a), hence, headaches induced by hypoxia or hypercapnia related to COVID-19 cannot be ruled out (Belvis, 2020); and (c) activation of the trigeminal vascular system, which is a crucial factor in the development of migraines (Pietrobon and Striessnig, 2003). Studies have suggested that COVID-19 vaccines can cause vascular and neural damage through an immune-inflammatory response mediated abnormal activation of the trigeminal vascular system (Caronna et al., 2020; Tana et al., 2022b; Consoli et al., 2022; Folegatti et al., 2020), hence, the impact of vaccination is also a significant consideration. Thirdly, a possible nocebo effect in migraine patients, mediated by negative health expectations, has been observed frequently in relation to therapeutic efficacy (Mitsikostas, 2016; Mitsikostas et al., 2020), and this has been substantiated by research conducted by Zevallos-Vásquez et al. (2023). Regardless of whether the increase in migraine occurrences during the pandemic was due to medication use or other causes, this led to confusion between common headache incidences and medication-related migraines, ultimately reducing the number of reported cases. While the post-COVID increase in reports of medication-related migraines does not significantly differ from previous years, recent studies have indicated a higher risk of developing migraines post-COVID (Xu et al., 2022), potentially due to organic brain damage caused by the virus (Douaud et al., 2022). The extent to which these impacts will continue over time requires further extensive clinical trials and data analysis to determine.

In the pharmacological categorization of drugs, immunosuppressants are identified as one of the principal high-risk categories. Despite the absence of systematic reviews in PubMed that report on the association between this class of drugs and migraine, individual searches for drugs within this category reveal multiple studies supporting such an association. For instance, in our study, Ozanimod, a novel therapeutic agent for multiple sclerosis, exhibited a significantly higher incidence of headache adverse events in a multicenter, randomized controlled trial compared to the interferon β-1a control group (Comi et al., 2019). Similarly, Apremilast, used in the treatment of psoriasis, has been reported in related research to cause headache-related adverse reactions (Bissonnette et al., 2016; Aljefri et al., 2022). Furthermore, drugs such as Fingolimod and Belimumab have also been documented in clinical studies for their association with migraine. These findings corroborate the conclusion of this study that migraine-related adverse events are a common potential complication of immunosuppressants. Although this study identified only five immunosuppressants with a higher association with migraine, it underscores the importance of vigilance regarding adverse reactions when employing immunosuppressants in the future.

Estrogens and progestogens, well-known inducers of migraine, are widely used in medical abortions and the alleviation of menopausal syndrome. Research indicates that the natural decline in luteal phase estrogen during the latter part of the menstrual cycle is one of the natural triggers for migraine (DrugBank, 2025), while high concentrations of estrogen can also induce migraine (Machado et al., 2010). Generally, estrogenic drugs are employed to mitigate menopausal syndrome, and a study by Kaiser HJ et al. has confirmed that hormone replacement therapy can induce drug-related migraine adverse events (Kaiser and Meienberg, 1993). These adverse events may occur due to a reduction in blood medication levels following hormone replacement therapy or simply due to excessive dosage, leading to migraine attacks with aura. Progestogens, commonly used as emergency contraceptives, have been established in previous research to cause migraine, potentially due to their complex interactions with estrogen, mimicking the effect of a decline in luteal phase estrogen levels. In summary, when clinically employing these hormonal medications, particular attention should be paid to the dosage, especially for patients with a history of migraine, as even slight oversight can provoke severe adverse reactions.

The phenomenon of antidepressant-induced adverse reactions, particularly drug-related migraines, has not been extensively documented in prior research and represents a novel finding in the current study. The potential causality may stem from the proclivity of antidepressant medications to alleviate symptoms associated with anxiety and depression, such as headaches. The absence of substantial literature on this topic may have contributed to an underestimation of the potential risks associated with drug-related migraines. To substantiate these preliminary findings, further fundamental research, comprehensive clinical trials, and extensive data analysis are imperative. Meanwhile, it is advisable to enhance vigilance and prevention against the adverse reactions of drug-related migraines.

In this study, the pharmaceutical agents most strongly associated with migraine incidence include lorcaserin (BCPNN = 3.33), tasimelteon (BCPNN = 3.2), and botulinum toxin type A (BCPNN = 3.06). These findings necessitate enhanced vigilance for migraine symptoms in patients prescribed these medications, particularly in those with predisposing factors. This connection is detailed in Table 4. Comprehensive discussions and analyses are conducted on these medications and their respective categories. Lorcaserin, a highly selective agonist for the 5-hydroxytryptamine 2C (5-HT_2_C) receptor, is primarily used for appetite suppression and obesity management (Hoy, 2013). Although current evidence does not directly link the 5-HT_2_C receptor with migraines or the trigeminal vascular system, several clinical trials have reported migraines as a common adverse reaction (Smith et al., 2010; Fidler et al., 2011), suggesting a potential role for 5-HT_2_C in pharmacologically induced migraines. Additionally, this study identifies serotonin-norepinephrine reuptake inhibitors (SNRIs) such as desvenlafaxine and milnacipran as related to migraines, further emphasizing the significance of the 5-HT pathway in the pathophysiology of migraines. Tasimelteon, an agonist targeting the MT1 and MT2 receptors, is employed in the treatment of Non-24-Hour Sleep-Wake Disorder, with studies reporting headaches as an adverse reaction (Yu et al., 2000). Research over the past decades suggests that melatonin exerts analgesic effects through the MT2 receptor (Yoon et al., 2008; Arreola-Espino et al., 2007; Yu et al., 2000; Ambriz-Tututi and Granados-Soto, 2007), and agonist-induced desensitization of the MT2 receptor may diminish this effect (Witt-Enderby et al., 2003; Dubocovich and Markowska, 2005). Furthermore, MT2-mediated vasodilation could also play a role in the onset of migraines (Doolen et al., 1998). Ramelteon, also within this category, has been shown to be associated with migraines, indicating that melatonin receptor agonists may have a potential risk of inducing migraines. Botulinum toxin type A, widely used in cosmetic applications since its FDA approval in 2002, is also employed in the treatment of migraines. However, the FAERS and product monographs indicate that it may trigger migraines. According to research by Silberstein S, a small dose (25 units) may alleviate migraine symptoms, whereas a higher dose (75 units) proves ineffective (Silberstein et al., 2000), suggesting that the migraine-inducing potential of this toxin may be dose-dependent. Clinically, it is crucial to control dosage to minimize the occurrence of drug-related migraines.

This study is not devoid of limitations. Firstly, although disproportionality analysis is instrumental in identifying potential drug-related adverse events, it lacks the inherent ability to establish causality. Given the observational nature of the FAERS database, combined with the absence of a randomized design, drawing definitive causal inferences is problematic. Furthermore, controlling for confounding factors, such as age, gender, patient demographics, severity of illness, duration of treatment, comorbid conditions, and concomitant medications, is challenging in such observational studies, thereby complicating the interpretation of results. A significant majority (70.6%) of the FAERS samples originate from the United States, limiting the generalizability of the study findings; moreover, the voluntary nature of the FAERS data introduces biases such as underreporting or overreporting, which affect the accuracy of the data and the generalizability of the results. The frequent absence of critical demographic information in reports renders risk assessment in specific subpopulations more challenging. Moreover, the inability to know the total number of patients exposed to a particular drug limits accurate estimation of adverse event incidence, a calculation crucial for authentic risk assessment. To overcome these limitations, future research should prioritize well-designed prospective cohort studies and randomized controlled trials to establish a stronger causal link between drugs and migraine. Such studies would enable more precise risk assessments across different underlying disease backgrounds and further explore how drugs interact with pre-existing conditions to influence migraine risk. Additionally, integrating complementary data sources such as electronic health records (EHRs) and medical insurance claims could mitigate the inherent biases of spontaneous reporting systems and provide a more comprehensive understanding of drug dosages, duration of treatment, and their use in real-world clinical settings. Additionally, as our current study assessed the correlation between drugs and migraine through disproportionality analysis without involving some clinical drug data, further external validation is essential. Ultimately, conducting large-scale clinical trials is crucial for validating existing findings, elucidating the mechanisms of drug-related adverse events, and refining clinical practice guidelines, thereby ensuring that treatment decisions are based on the most reliable evidence, significantly enhancing patient safety and treatment outcomes.

## Conclusion

In summary, this study leveraged real-world data on adverse drug reactions and employed disproportionality analysis to identify 39 medications potentially associated with drug-related migraine. The medications identified were categorized based on their BCPNN values to assess risk levels. The findings delineate the epidemiological characteristics of drug-related migraine and highlight potentially high-risk medications (lorcaserin, tasimelteon, botulinum toxin). This research provides reliable data for clinical decision-making aimed at mitigating the risk of such adverse reactions. Importantly, the study clarifies the complex relationship between medication usage and the incidence of migraines, underscoring the importance of understanding drug safety. It calls for increased vigilance regarding the newly identified high-risk medications and the initiation of further clinical studies to more deeply explore the adverse effects of these drugs.

## Data Availability

Publicly available datasets were analyzed in this study. This data can be found here: https://www.fda.gov/drugs/drug-approvals-and-databases/fda-adverse-event-reporting-system-faers-database.
